# Two Lysines in the Forkhead Domain of Foxp3 Are Key to T Regulatory Cell Function

**DOI:** 10.1371/journal.pone.0029035

**Published:** 2012-01-11

**Authors:** Yujie Liu, Liqing Wang, Rongxiang Han, Ulf H. Beier, Wayne W. Hancock

**Affiliations:** 1 Laboratory of Medicine, Division of Transplant Immunology, Department of Pathology, School of Medicine, University of Pennsylvania, The Children's Hospital of Philadelphia, Philadelphia, Pennsylvania, United States of America; 2 Division of Nephrology, Department of Pediatrics, School of Medicine, University of Pennsylvania, The Children's Hospital of Philadelphia, Philadelphia, Pennsylvania, United States of America; Institut Jacques Monod, France

## Abstract

**Background:**

The forkhead box transcription factor, Foxp3, is master regulator of the development and function of CD4+CD25+ T regulatory (Treg) cells that limit autoimmunity and maintain immune homeostasis. The carboxyl-terminal forkhead (FKH) domain is required for the nuclear localization and DNA binding of Foxp3. We assessed how individual FKH lysines contribute to the functions of Foxp3 in Treg cells.

**Methodology/Principal Findings:**

We found that mutation of FKH lysines at position 382 (K17) and at position 393 (K18) impaired Foxp3 DNA binding and inhibited Treg suppressive function *in vivo* and *in vitro*. These lysine mutations did not affect the level of expression of Foxp3 but inhibited *IL-2* promoter remodeling and had important and differing effects on Treg-associated gene expression.

**Conclusions/Significance:**

These data point to complex effects of post-translational modifications at individual lysines within the Foxp3 FKH domain that affect Treg function. Modulation of these events using small molecule inhibitors may allow regulation of Foxp3+ Treg function clinically.

## Introduction

CD4+CD25+ T-regulatory (Treg) cells are important to the maintenance of immunological homeostasis and self-tolerance [1], [2]. The forkhead box transcription factor, Foxp3, is now recognized as the master regulator of the development and function of CD4+CD25+ Treg cells [3], [4], [5]. Deletion or mutations of Foxp3 cause lethal autoimmunity in *scurfy* mice and profound morbidity in patients suffering from IPEX (immune dysregulation, polyendocrinopathy, enteropathy, X-linked) [5], [6]. In contrast, retroviral transduction of CD4^+^ T cells with Foxp3 induces a Treg phenotype and the capacity to suppress lymphocyte proliferation [3]. Murine and human Foxp3 protein share a high degree of homology, with 429 and 431 amino acids, respectively. Both proteins contain a repressor domain, a zinc finger domain, a leucine zipper domain, and a conserved DNA binding C-terminal forkhead domain (FKH) important for the nuclear translocation and DNA binding of Foxp3 [7], [8]. Mutations in the FKH and leucine zipper domains are associated with clinical autoimmunity [9], [10], [11], [12], indicating the importance of these regions. For example, mutations within the FKH domain that disrupt the interaction of Foxp3 and NFAT result in loss of the ability of Foxp3 to downregulate IL-2 expression and upregulate CTLA4 and CD25 expression [13]. Foxp3 also inhibits IL-2 expression by interacting with AML1/Runx1, which is normally an activator of IL-2 expression. Three amino acids located immediately N-terminal to the FKH domain are important for this inhibition, and their mutation (D329V, Y330H, and K332L) impaired Treg suppressive function [14]. Similarly, a single glutamic acid mutation (E251) or deletion of E250 in the leucine-zipper domain inhibited Foxp3 dimerization and abrogated its repressor functions [7], [15].

Studies from mice and human demonstrated that the histone acetyltransferases (HAT), Tat-interactive protein (Tip60) and p300, increase lysine (K) ε-acetylation of Foxp3, as does use of histone/protein deacetylase inhibitors (HDACi) [16], [17], [18]. Interestingly, Foxp3 acetylation promotes Treg function by increasing DNA binding to several promoters, including that of *IL-2* [16], as well as by increasing the resistance of Foxp3 to polyubiquitination and proteasomal degradation [18], [19]. Cytokine-dependent signals play central roles in promoting the DNA binding of Foxp3. Thus, TGFβ increases binding of acetylated human Foxp3 to the *IL-2* promoter, whereas TGFβ and IL-6 together decrease this binding, and binding is restored by treatment with an HDACi [20]. While the contributions of individual lysines have not been determined, it is clear that acetylation of Foxp3 plays an important role in the regulation of Foxp3 production and function in Treg cells.

In the current study, we found that mutations of single lysines in the FKH domain of Foxp3, Lys17Arg (K17R) or Lys18Arg (K18R), affected Foxp3 DNA binding, impaired Treg suppressive function *in vitro* and *in vivo*, and altered expression of Treg-associated markers, cytokines, and levels of HAT and HDAC enzymes. Our data suggest that point mutations in Foxp3 can have different effects than the widely recognized mutations that lead to the development of autoimmunity in scurfy mice and patients with IPEX syndrome. The complex effects of these mutations point to the importance of FKH lysine residues in control of Foxp3 function.

## Results

### C-terminal Foxp3 lysine mutations affect Treg suppressive function and gene expression

**Figure 1:**
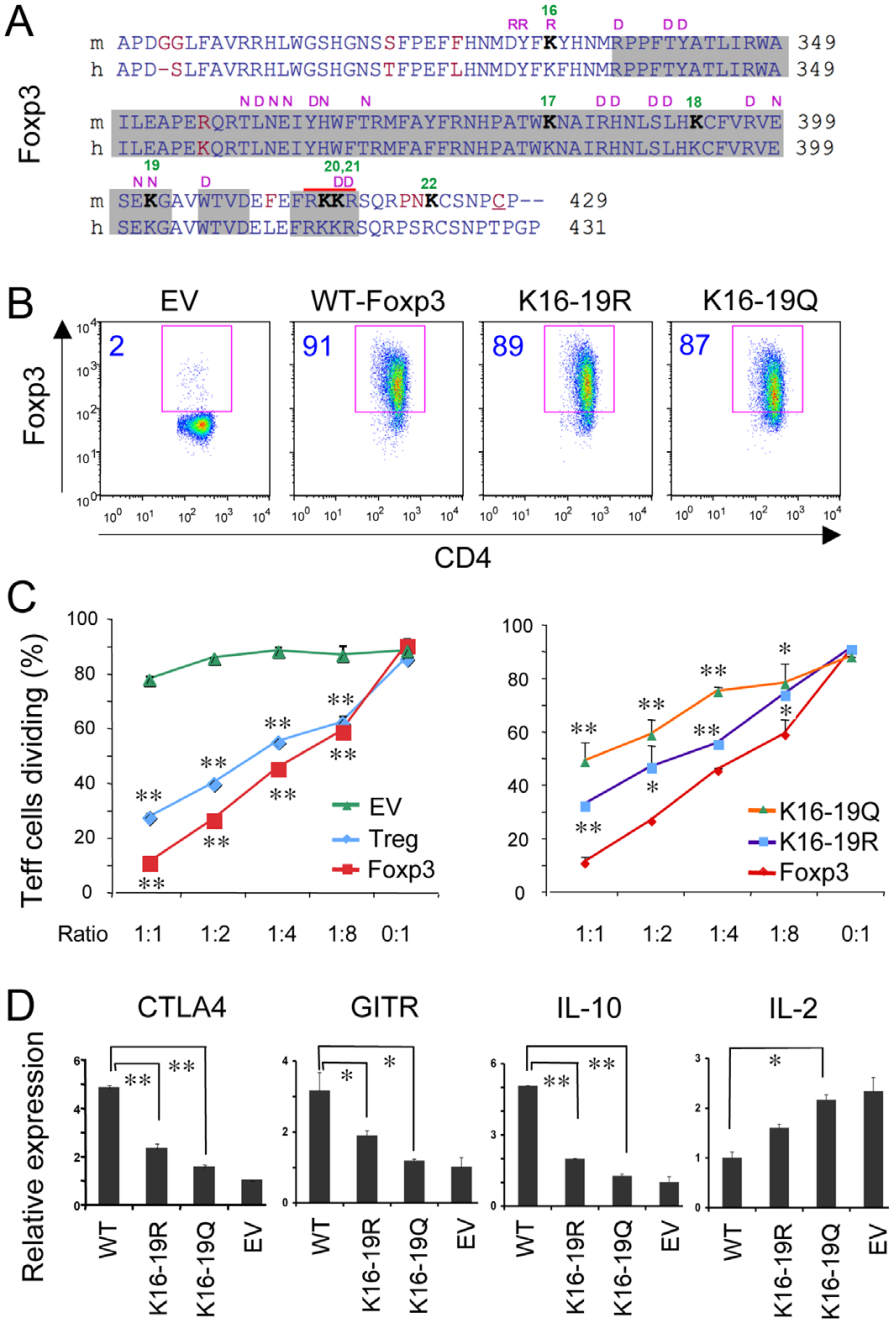
Foxp3 lysine mutations affect Treg suppressive function and gene expression. (A) Comparison of amino acid sequences of the C-terminal regions of mouse (m) and human (h) Foxp3, with FKH domains in gray. Lysine at position 332 is K16 and the FKH domain contains K17–K21 (consecutive lysines numbered in green), with residues important for Runx1 (R), DNA (D) or NFAT (N) binding indicated in purple and non-conserved residues shown in red; adapted from [16]. (B) CD4^+^CD25^−^ T cells were transduced with retroviruses encoding WT Foxp3, Foxp16-19R, Foxp16-19Q or EV; Foxp3 staining showed >85% transduction efficiency (%transduced cells shown in blue in each panel). (C) In vitro Treg suppression assays in which 5×10^5^ CFSE-labeled Teff cells were stimulated for 72 h with CD3 mAb in the presence of 5×10^5^ irradiated APC and the indicated ratios of Treg to Teff cells. Data are mean ± SD of duplicate measurements of the percentages of dividing Teff cells, and results are representative of 3 independent experiments; *p value<0.05, **p<0.01 compared to empty vector (EV) in left panel or compared to WT Foxp3 in right panel. (D) RNA derived from CD4^+^CD25^−^ T cells transduced with WT Foxp3, Foxp3 K16-19R, K16-19Q or EV were analyzed for CTLA4, GITR, IL-2, and IL-10 gene expression by qPCR and data were normalized to 18S; *p value<0.05, **p<0.01 compared to WT Foxp3. Graphs show means ± SD and results are representative of 3 independent experiments.

Five FKH domain lysines (K17, 18, 19, 20, and K21) are conserved between humans and mice (**Fig. 1A**). K17 and 18, but not K20, are important for Treg suppressive function in vitro [16], whereas K20 and K21 are important for Foxp3 localization within the nucleus [7]. An additional lysine, K16, adjacent to the FKH domain, is important for the interaction of Foxp3 with AML/RunX1 [14]. We therefore focused on K16, 17, 18 and 19 and investigated whether these residues were important for Foxp3 function. We used site-directed mutagenesis to substitute the 4 residues (K16-19) with arginine (R), so as to prevent acetylation but conserve the positive charge, or with glutamine (Q), so as to change the positive charge of lysine to neutral and possibly mimic features of acetylation [21]. Wild-type mouse Foxp3 (WT-Foxp3) or mouse Foxp3 constructs containing mutated K16-19R or K16-19Q were cloned into a retroviral vector (MinR1) encoding nonfunctional nerve growth factor receptor (NGFR), MinR1 constructs were transfected into packaging cell lines, and supernatants from packaging cell lines were used to infect activated mouse primary CD4+ T cells. Flow cytometric analysis (**Fig. 1B**) indicated transduction efficiencies of >85% for mutant and WT Foxp3, and comparable levels of Foxp3 expression, indicating the mutations did not affect Foxp3 expression in Treg cells.

We next examined whether these Foxp3 mutations affected Treg suppressive function. Mouse primary CD4+ T cells transduced with vectors encoding the WT-Foxp3 exhibited comparable or better suppressive function than native CD4+CD25+ Treg cells isolated from C57BL/6 mouse (B6-Treg), whereas empty-vector (EV) transduction essentially lack any suppressive function (**Fig. 1C**, left panel). However, compared with transduction of CD4+ T cells with vectors encoding WT-Foxp3, transduction of vectors containing lysine mutations (K16-19R or K16-19Q) led to markedly impaired Treg suppressive ability, with glutamine mutations (K16-19Q) displaying more impaired function (**Fig. 1C**, right panel). These data indicated these four lysines (K16-19) are important for Treg suppression function, at least *in vitro*.

We used qPCR to examine the mRNA levels of several genes of functional significance for Tregs [22], [23], [24], [25], [26], [27]. Compared with WT-Foxp3 transduced CD4+ T cells, CD4+ T cell transduction with vectors containing Foxp3 K16-19R or K16-19Q showed lower expression of cytotoxic T lymphocyte-associated antigen 4 (CTLA-4), glucocorticoid-induced tumor necrosis factor-receptor-related protein (GITR), and IL-10, with K16-19Q mutations showing even lower mRNA expression than K16-K19R mutations (**Fig. 1D**). Moreover, compared with WT-Foxp3-transduced CD4+ T cells, CD4+ T cells transduced with Foxp3-K16-19R or K16-19Q exhibited a higher level of IL-2 (**Fig. 1D**), indicating that these four lysines are likely involved in regulating expression of key Treg genes. Since the mutation of lysine to glutamine, which mimics acetylation in some systems but not others [28], unexpectedly impaired Treg function to a greater extent than arginine mutations, we focused our studies on the arginine mutants that maintain the same polarity as lysine.

### Single Foxp3 residues, K17 or K18, are important for Treg suppressive function and gene expression

**Figure 2:**
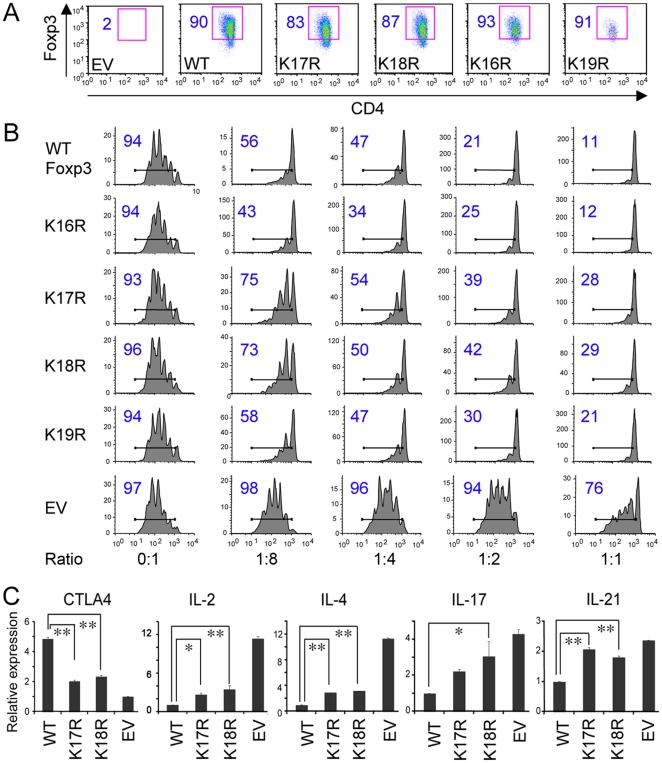
Single Foxp3 lysine mutations affect Treg suppressive function and gene expression. (A) CD4+ CD25− T cells transduced with retroviruses encoding WT Foxp3, K16R, K17R, K18R, K19R or EV; Foxp3 staining showed >80% transduction efficiency. (B) Effects of single lysine mutations on Treg suppressive activity. (C) RNA derived from CD4+CD25− T cells transduced with WT Foxp3, K17R, K18R or EV were analyzed for CTLA4 (in the absence of CD3/CD28 mAbs) and IL-2, IL-4, IL-17 and IL-21 (in the presence of CD3/CD28 mAbs) gene expression by qPCR. Data were normalized to 18S; *p<0.05, **p<0.01 compared to WT Foxp3. Graphs show means ± SD and results are representative of 3 independent experiments.

We tested the roles of individual lysines in Treg function by transducing mouse primary CD4+ T cells with MinR-Foxp3 constructs containing K16R, K17R, K18R or K19R. Transduction efficiency in each case was >80% as indicated by Foxp3 expression (**Fig. 2A**), and there were no significant differences between mutants. However, Treg suppression assays showed that compared with CD4+ T cells transduced with WT-Foxp3, cells transduced with Foxp3 K17R or K18R mutant exhibited significantly impaired Treg suppression (**Fig. 2B**), whereas Foxp3 K16R increased Treg suppressive function at certain Treg to Teff cell ratios, and K19R mutation did not impair Treg suppression. Additionally, CD4+ T cells transduced with Foxp3 K17Q, K18Q, K17E, or K18E mutant also resulted in impaired Treg suppressive function to varying extents (data not shown). These data thereby suggested that K17 and K18 of the Foxp3 FKH domain were especially important for Treg suppressive function *in vitro*. In addition, compared with CD4+ T cells transduced with WT-Foxp3, CD4+ T cells transduced with Foxp3 K17R or K18R mutant exhibited markedly decreased mRNA level of CTLA-4 and slightly decreased levels of GITR, CD62L, and IL-10 (**Fig. 2C** and data not shown). Foxp3 K17R or K18R mutants showed significantly increased levels of IL-2, IL-4, IL-17, and IL-21 upon stimulation of CD3/CD28 mAbs (**Fig. 2C**). These observations demonstrated that the K17 and K18 residues contribute to Foxp3-mediated control of cytokine expression within Treg cells and to maintenance of Treg suppressive function.

### K17 and K18 mutants exhibit impaired Foxp3 DNA binding ability

**Figure 3:**
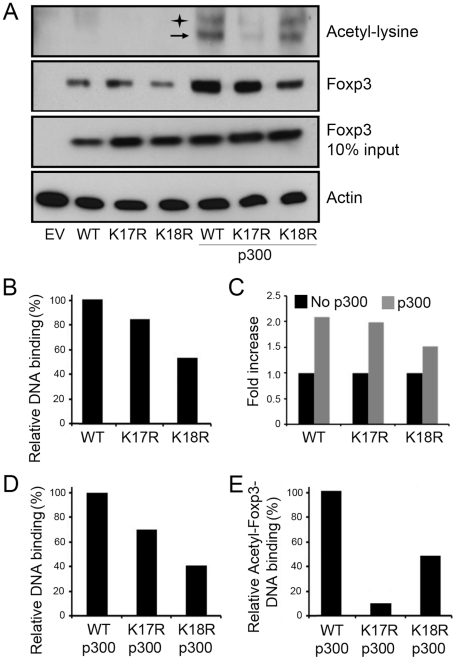
Foxp3 mutants impair Foxp3 DNA binding ability. 293T cells were transfected with EV, WT Foxp3, K17R or K18R without or with p300 expression vectors, and 48 h later, cell lysates were harvested. (**A**) Equal amounts of cell lysates were incubated with biotin-labeled Foxp3 binding site nucleotide, and Foxp3 DNA binding was detected with anti-Foxp3 or anti-acetyl-lysine Abs. The protein expression levels of Foxp3 and loading control β-actin were detected by western blotting; arrow indicates acetylated Foxp3 bound to DNA, and star indicates non-specific binding. (**B–D**) The densities of Foxp3 DNA-binding bands were measured using Image-J software and normalized with Foxp3 input levels. (**B**) The relative Foxp3 DNA binding ability in the absence of p300 is shown. (**C**) Foxp3 and mutant DNA binding ability was increased in the presence of p300. (**D**) Comparison of relative Foxp3 DNA binding between WT and mutants in the presence of p300 is shown. (**E**) Comparison of relative acetylated Foxp3 binding level between WT and mutants is shown. Results are representative of 2 independent experiments.

While p300 is known to acetylate Foxp3 [18], the single point mutations (K17R, K18R) did not reduce overall Foxp3 protein acetylation (data not shown). However, as the C-terminal FKH domain mediates Foxp3 DNA binding [29], [30], we next compared the DNA binding of WT Foxp3 and mutants. Biotin-labeled Foxp3 binding site nucleotide [30] was incubated with cell lysates from 293T cells expressing WT or mutant Foxp3, with or without p300. DNA-bound biotinylated Foxp3 nucleotide was precipitated using streptavidin beads and detected by immunoblotting with anti-Foxp3 mAb. Compared to WT Foxp3, K18R had markedly decreased DNA binding ability, whereas K17R binding was only modestly decreased (**Fig. 3A and B**). Since transcription factor DNA binding ability is frequently increased by acetylation [31], we further investigated Foxp3 DNA binding ability after acetylation by p300. After adding p300, the DNA binding ability of both WT Foxp3 and mutants was increased up to two-fold, with a greater effect on K17R than K18R (**Fig. 3A and C**). Remarkably, the presence of p300 enhanced the differences between Foxp3 DNA binding of WT and mutant Foxp3, revealing a more than two-fold decrease in K18R mutant Foxp3 binding (**Fig. 3B and D**). We also assessed the level of acetylated-Foxp3 bound to DNA level by immunoblotting with anti-acetyl-lysine antibody after nucleotide pull-down of Foxp3, and found that K17R mutant had markedly decreased acetylated-Foxp3 bound to DNA (**Fig. 3A and E**). These data suggest that K17 and K18 contribute to regulation of Foxp3 DNA binding.

### FKH K17 and K18 residues regulate *IL-2* promoter acetylation and HDAC and HAT expression

**Figure 4:**
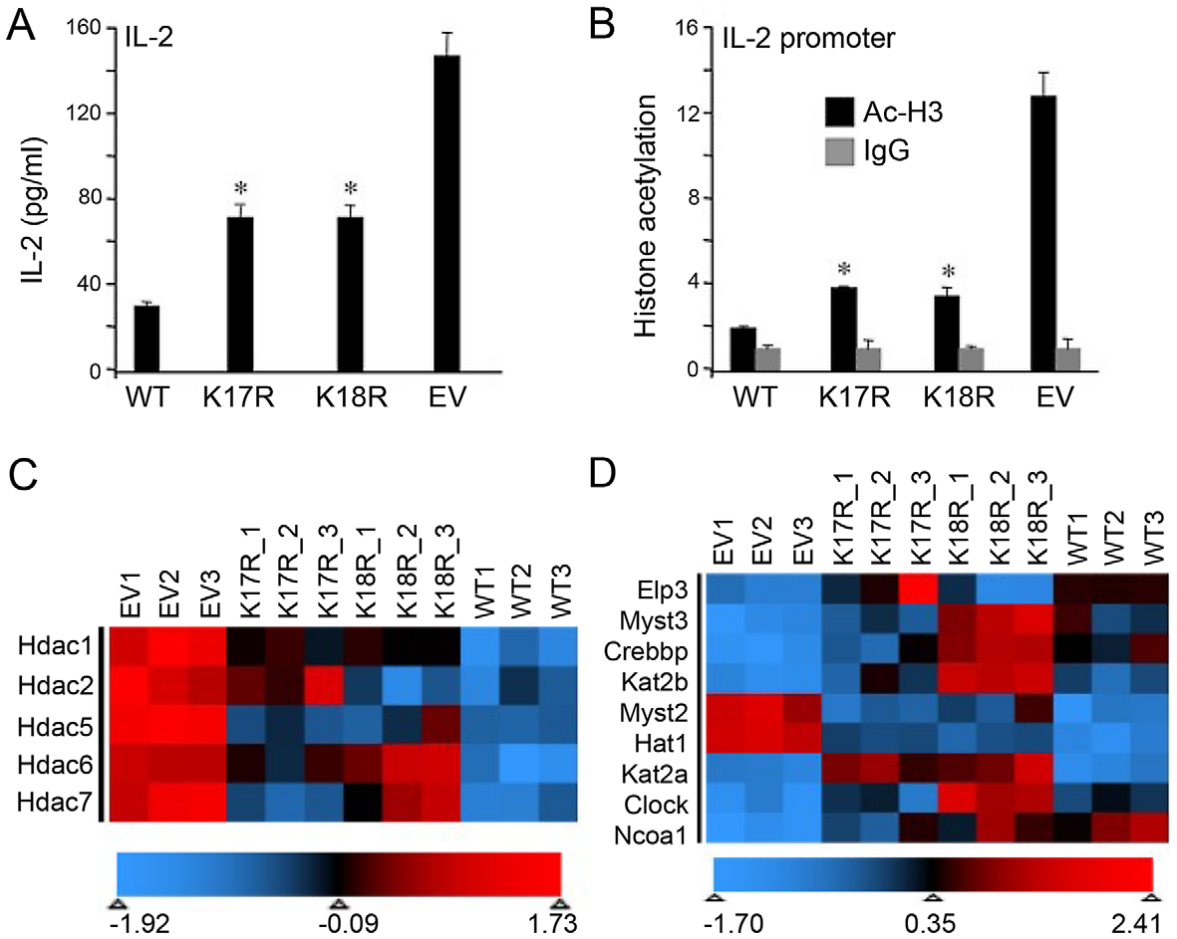
Foxp3 mutations affect chromatin remodeling and HDAC and HAT expression. CD4+ T cells transduced with WT Foxp3, K17R, K18R or EV were cultured in medium with CD3/CD28 mAb for 20 h. (**A**) IL-2 levels were detected by ELISA. (**B**) Chromatin extracts were precipitated with anti-AcH3 Ab or control IgG, and probed for the promoter regions of IL-2 and genes levels were determined by qPCR. Results (mean ± SD) in panels A and B are representative of 3 independent experiments, and *p<0.05, **p<0.01 compared to WT Foxp3. Heat maps indicating distinct expression profiles of genes encoding (**C**) HDAC and (**D**) HAT enzymes. Microarray experiments were performed using whole-mouse-genome oligoarrays (Mouse430a; Affymetrix), and array data were analyzed using MAYDAY 2.12 software [45]. Array data were subjected to robust multiarray average normalization. Normalized data were used for calculating fold changes of up- and downregulated genes using Student's test, and data with >2x differential expression (p<0.05 with Storey's FDR<0.1) were included in the analysis. Data underwent z-score transformation for display.

Foxp3 can bind to the *IL-2* promoter, in both natural Tregs and CD4+ T cells transduced with Foxp3 [13], at a site adjacent to the ARRE-2 NFAT/AP-1 response element [13], [32], [33]. Given the reduced DNA binding of our Foxp3 mutants, we measured IL-2 protein levels in supernatants of T cells transduced with WT Foxp3, K17R or K18R Foxp3, or EV. Both K17R and K18R mutations resulted in greater production of IL-2 than cells transduced with WT Foxp3 (**Fig. 4A**). In addition, since Foxp3 controls Treg function by competing with AP1 and cooperatively binding with NFAT [13], we assessed if K17R and K18R mutations affected Foxp3 targeting of the NFAT∶AP1 complex. We transfected 293T cells with 3x NFAT∶AP1-IL-2-Luc reporter, plus expression vectors for NFAT and WT or mutant Foxp3 (K17R, K18R). WT Foxp3 significantly inhibited NFAT transcriptional activity, consistent with previous studies [13]. Both K17R and K18R Foxp3 mutants showed similar impairment of NFAT-mediated transactivation, suggesting that Foxp3 K17 and K18 are not involved in Foxp3-NFAT-DNA formation (**Figure S1**). Foxp3-mediated transcriptional activation and repression are associated with specific histone modifications [33], [34]; e.g. Foxp3 binding to the *IL-2* promoter leads to histone H3 deacetylation and repressive chromatin remodeling. Accordingly, we assessed acetylated histone 3 (Ac-H3) at the *IL-2* promoter using CD4+ T cells transduced with WT or mutant Foxp3 (**Fig. 4B**). A significant amount of Ac-H3 remained at the *IL-2* promoter of CD4+ T cells transduced with empty vector. However, transduction with WT Foxp3 markedly reduced Ac-H3 binding to the *IL-2* promoter, whereas cells transduced with mutant Foxp3 (K17R and K18R) exhibited increased Ac-H3 binding to the *IL-2* promoter (**Fig. 4B**). These data indicate that K17 and K18 are involved in Foxp3-mediated remodeling of *IL-2* chromatin structure in CD4+ T cells. In addition to recruiting HDAC and HAT enzymes to target genes such as *IL-2*, Foxp3 could conceivably regulate the expression of these enzymes in Treg cells. Consistent with this concept, we found that transduction with WT versus Foxp3 mutants led to differential expression of HDAC and HAT genes. Compared to EV control, transduction of WT Foxp3 led to significant suppression of HDAC 1, 2, 5, 6, and 7 mRNA. In contrast, mutant Foxp3 transduction resulted in less suppression of HDAC expression (**Fig. 4C**), and considerably greater HAT expression (**Fig. 4D**). Collectively, these data indicate that K17 and K18 contribute to Foxp3-dependent chromatin remodeling and modulation of HDAC and HAT expression.

### K17 and K18 are important for Foxp3 suppressive function *in vivo*

**Figure 5:**
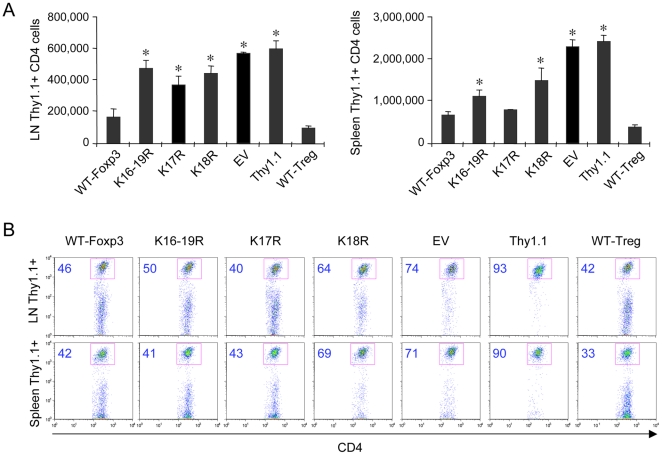
Foxp3 mutants impair Treg function in vivo. (**A**) 1×10^6^ Thy1.1+ CD4+CD25− T cells were co-transferred with 1×10^6^ Thy1.2+ CD4+ T cells transduced with WT Foxp3, K16-19R, K17R, K18R or EV, or with purified normal B6 Treg cells, into Rag1−/− mice. At 7 d post-transfer, single-cell suspensions from lymph node or spleen samples were stained for FACS analyses; the numbers (**A**) or percentages (**B**) of CD4+ Thy1.1+ cells are shown. Results are representative of 2 independent experiments, and *p<0.05 compared to WT Foxp3.

Since Treg cells play an important role in the control of homeostatic proliferation [27], [35], [36], [37], we evaluated the effects of FKH lysine mutations on this process. We adoptively transferred into immunodeficient C57BL/6 Rag1−/− mice one million Thy1.1+ CD4+CD25− cells alone, or along with one million Thy1.2+ CD4+ T cells transduced with EV, WT Foxp3, Foxp3 K16-19R, Foxp3 K17R or K18R, or along with one million native Treg cells isolated from C57BL/6 mice. On day 7 post-transfer, we enumerated cells from the lymph nodes (LN) and spleen of each mouse. The co-transfer of CD4+ T cells transduced with WT Foxp3, or native Tregs, led to substantially decreased homeostatic T cell proliferation of Thy1.1+ CD4+CD25− cells compared with the results of transfer of Thy1.1+ CD4+CD25− alone, or cotransfer of Thy1.1 CD4+CD25− cells transduced with EV (**Fig. 5A**). However, adoptive transfer of Thy1.1+ CD4+CD25− cells, plus CD4^+^ T cells transduced with mutant Foxp3 K17R or Foxp3 K18R, led to impaired suppression of homeostatic T cell proliferation as compared with cotransfer of Thy1.1+ CD4+CD25− cells plus CD4+ T cells transduced with WT Foxp3 (**Fig. 5A**). We also evaluated the percentage of Thy1.1+ CD4+CD25− cells among these groups (**Fig. 5B**). Transfer of Thy1.1+ CD4+CD25− alone exhibited the highest percentage of Thy1.1+ cells, followed by cotransfer with EV, and then Foxp3 K18R. However, there was no statistically significant difference between the other groups, including cotransfer with WT Foxp3, B6 Treg, Foxp3 K16-19R or Foxp K17R. Thy1.1+ CD4+ T cells cotransfered with CD4+ T cells transduced with Foxp3 K18R displayed significantly higher cell numbers than those of Foxp3-K17R, consistent with our finding that Foxp3 K18R has lower DNA binding ability (**Fig. 3**). These data showed that the 2 lysine residues are important for Treg *in vivo* suppression of lymphopenia-induced expansion of CD4^+^ T cells. They also suggest that there are differences between the 2 Foxp3 mutants that might be reflected by differences in gene expression in corresponding Treg cells.

### FKH lysine mutations affect Treg gene expression profiles

**Figure 6:**
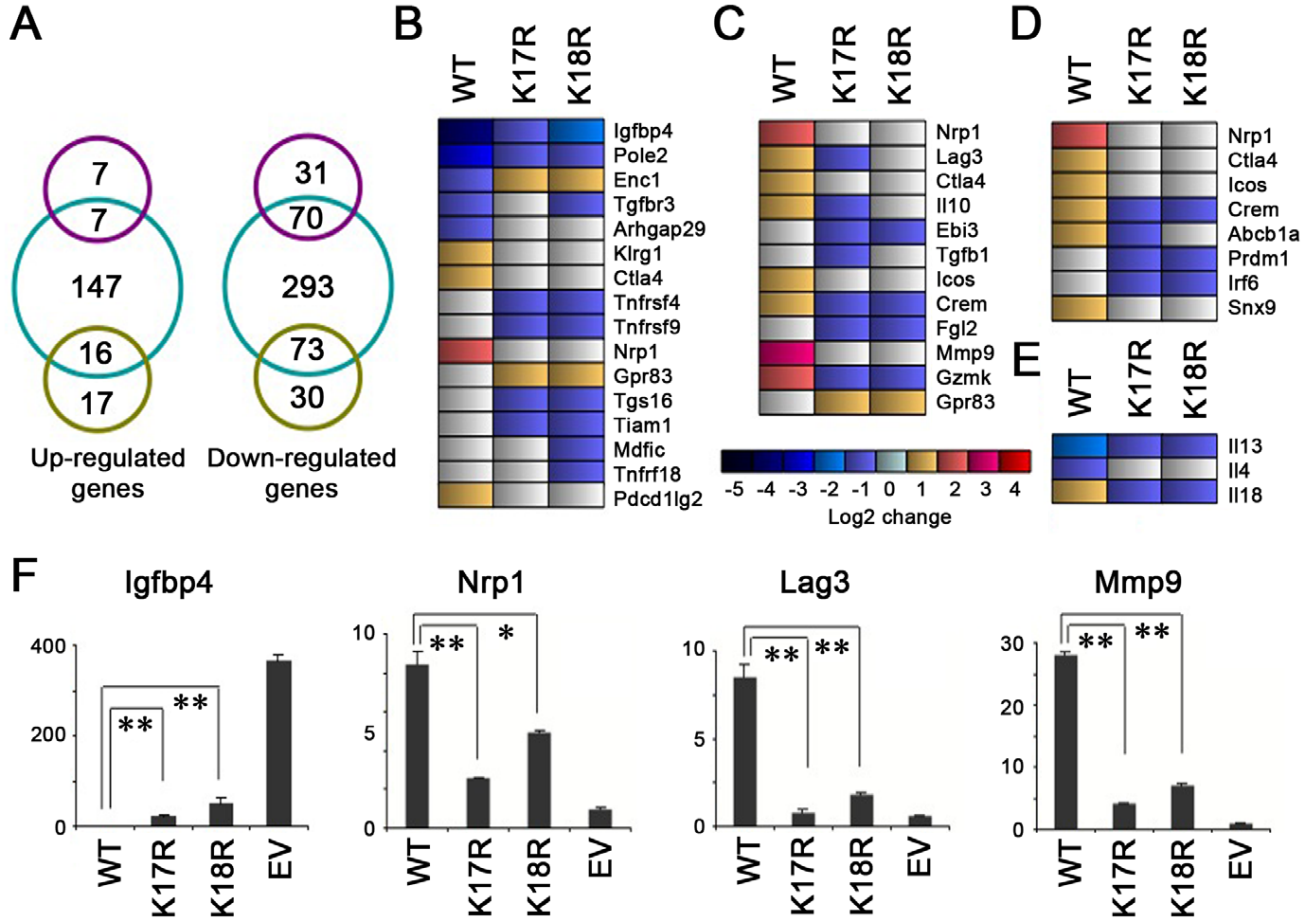
Comparison of gene expression profiles of CD4+ T cells transduced with WT Foxp3, K17R, K18R or EV. (**A**) Venn diagrams summarizing overlapping upregulated (left) or downregulated (right) gene expression profiles of CD4+ T cells transduced with WT Foxp3 (blue), K17R (purple) or K18R (yellow) compared with CD4+ T cells transduced with EV (cutoff was set as Log2 fold >1). (**B–E**) Heat maps showing gene expression profiles of CD4+ T cells transduced with EV, WT Foxp3, K17R or K18R. (**B**) Heat maps showing distinct Treg ‘signature’ gene expression profiles. (**C**) Heat maps indicating the change in gene expression profiles of Treg cell identified and putative suppressive genes. (**D**) Heat maps indicating the distinct gene expression profiles of Foxp3 directly bound genes. (**E**) Heat maps indicating distinct gene expression profiles of Treg-related cytokine genes. (**F**) qPCR assays of Treg-specific genes selected from microarray analyses; results are representative of 3 independent experiments, and *p<0.05, **p<0.01 compared to WT Foxp3.

To assess the possible effects of Foxp3 K17 and K18 mutations on Treg gene expression, global gene expression profiling was performed using Affymetrix 430 2.0 mouse oligonucleotide gene expression arrays. Normalized gene expression values of the CD4+ T cells transduced with EV were used as the reference for comparison with data from CD4+ T cells transduced with WT, K17R or K18R Foxp3. Compared with EV transduced CD4+ T cells, CD4+ T cells transduced with WT Foxp3 had 147 up-regulated genes, whereas CD4+ T cells transduced with Foxp3 K17R or K18R had only 14 or 33 up-regulated genes, respectively (Log2 >1). Among the up-regulated genes, 7 or 16, respectively, overlapped with WT Foxp3 (**Fig. 6A**). Conversely, CD4+ T cells transduced with WT Foxp3 had 293 down-regulated genes, Foxp3 K17R had 101 down-regulated genes, and Foxp3 K18R had 103 down-regulated genes (Log2<−1). Among the down-regulated genes, 70 or 73, respectively, were shared with WT Foxp3 (**Fig. 6A**). These data showed that a single lysine mutation (K17R or K18R) within the Foxp3 FKH domain markedly affects Foxp3-dependent gene expression, suggesting an important role of each lysine in controlling Foxp3+ Treg functions.

**Figure 7:**
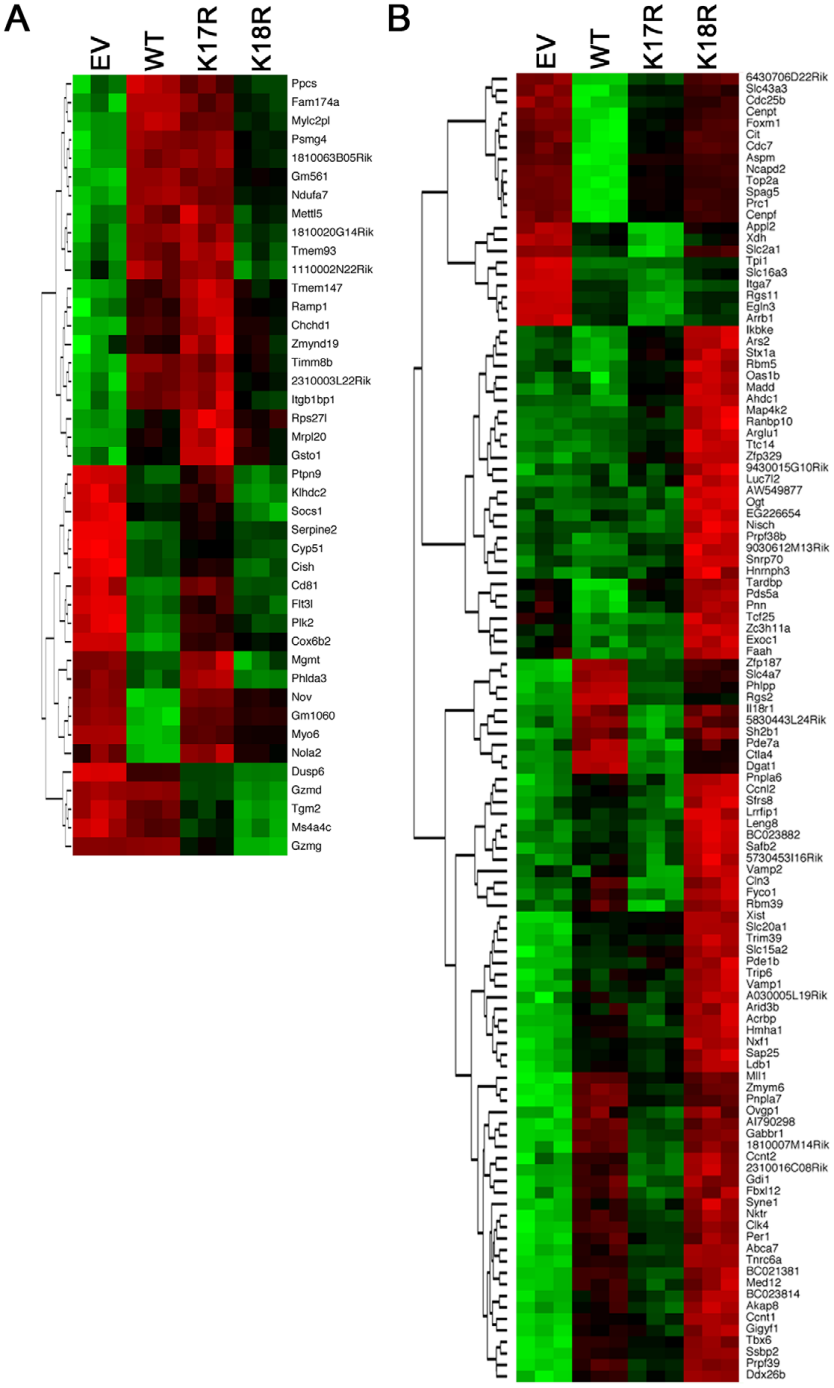
Comparison of gene expression profiles of CD4+ T cells transduced with Foxp3 K17R versus K18R. Heat maps showing distinct gene expression profiles of CD4+ T cells transduced with WT Foxp3, K17R, K18R or EV.

Further examination of the dataset resulted in several findings. First, compared with CD4+ T cells transduced with WT Foxp3, CD4+ T cells transduced with the 2 mutants had altered expression of multiple Treg ‘signature’ genes. WT Foxp3 transduction led to marked down-regulation of *Igfbp4*, *Pole2*, *Enc1*, *Tgfbr3* and *Arhgap29*, whereas expression of these genes was only modestly downregulated or increased in CD4+ T cells transduced with Foxp3 K17R and K18R (**Fig. 6B**). Conversely, *Nrp1*, *Gpr83*, *Rgs16*, *Tnfrsf4*, *Tnfrsf9*, *Klrg1*, *Mdfic*, *CTLA-4*, *Tiam1*, *Tnfrf18*, and *Pdcd1Ig2* were up-regulated in CD4+ T cells transduced with WT Foxp3 but were decreased in CD4+ T cells transduced with Foxp3 K17R and K18R (**Fig. 6B**). Second, we compared expression across the groups of known or putative suppressor genes found in Treg cells, including *Nrp1*, *Lag3*, *CTLA-4*, *IL-10*, *Epstein-Barr virus-induced gene 3 (Ebi3)*, *Tgfb1*, *ICOS*, *Crem*, *Fgl2*, *Mmp9*, *Gzmk*, and *Gpr83* (**Fig. 6C**). Except for *Grp83*, and in contrast to data from WT Foxp3, the expression of each of these genes was decreased in CD4+ T cells transduced with Foxp3 K17R or 18R. These data are consistent with the impaired suppressive function of CD4+ T cells transduced with either mutant as compared with that of CD4+ T cells transduced with WT Foxp3. Third, recent studies demonstrated that Foxp3 is able to directly bind to the promoters of a number of genes and up- or down-regulate their expression in Treg cells [34], [38]. Foxp3-dependent genes downregulated in CD4+ T cells transduced with Foxp3 K17R or K18R compared with WT Foxp3 included cell surface molecules: *Nrp-1*, *Abcb1a*, *CTLA-4* and *ICOS*; transcriptional factors: *Prdm1*, *Irf6* and *Crem*; and a vesicular trafficking gene: *Snx9* (**Fig. 6D**). Fourth, with respect to effects on cytokine gene expression, CD4+ T cells transduced with Foxp3 K17R or K18R exhibited higher levels of *IL-4* and *IL-13* and a lower level of *IL-18* compared with CD4+ T cells transduced with WT Foxp3 (**Fig. 6E**). These data suggest that each lysine residue may directly mediate Foxp3 binding to the binding sites on a range of target promoters. To validate the microarray results, we used quantitative PCR analysis to determine selected genes, including Igfbp4, Nrp1, Lag3 and Mmp9 (**Fig. 6F**); the results were consistent with the microarray results. Lastly, we compared the gene expression profile of CD4+ T cells transduced with Foxp3 K17R versus K18R (**Fig. 7**). In addition to the differences in expression of Treg ‘signature’ and suppressive genes, we found that CD4+ T cells derived from transduction of Foxp3 K18R were enriched for genes encoding cell division, cell cycle, and phosphoproteins (http://david.abcc.ncifcrf.gov/). These findings are consistent with our observations that CD4+ T cells transduced with K18R had weaker DNA binding (**Fig. 3**), and greater CD4+ Thy1.1+ T cell proliferation (**Fig. 5**), as compared with CD4+ T cells transduced with Foxp3 K17R. These data suggest that K17 and K18 have distinctive differences in the regulation of Foxp3+ Treg gene expression.

## Discussion

We previously demonstrated that dual substitutions of Foxp3 K17-18 with histidine residues led to impaired Treg suppressive function and inhibited Foxp3 DNA binding to the *IL-2* promoter [16]. We have now extended our studies to the use of mutations with greater structural similarity and focused on the effects of individual lysines of interest on the Treg phenotype. Our studies suggest that 2 lysine residues (K17 and K18) in the FKH domain of Foxp3 are important for Treg function *in vivo* and *in vitro*. Substituting these lysines with arginine impairs Treg suppressive function, alters Foxp3-mediated gene expression, diminishes Foxp3 DNA binding, and alters the chromatin remodeling state of target genes (e.g. *IL-2*). Although the 2 mutants impaired Treg function, they did not affect Foxp3 mRNA or protein level, indicating detection of Foxp3 mRNA or protein level is insufficient to indicate full functional capacity of Tregs. In addition, we compared the different gene expression profiles of CD4+ T cells transduced with WT, K17R or K18R Foxp3, and found significant differences between CD4+ T cells transduced with WT Foxp3 versus with mutant, as well as between the two mutants themselves, suggesting that the different lysines may play distinct roles in Foxp3 function.

Mutation of either K17R or K18R impaired the DNA binding of both unmodified and acetylated Foxp3, with K18R exhibiting a greater effect. There are at least 2 models of the interaction between acetylation and transcription factor DNA binding. First, acetylation can neutralize basic residues and promote transcription factor DNA binding, as was demonstrated in the current studies. In the presence of p300, the enhancement of DNA binding of K18R was lower than that of WT and K17R, suggesting that acetyl group on Foxp3 lysine residues may contribute to conformational changes during DNA recognition and binding. Second, DNA-binding dependent acetylation can occur, such as was demonstrated for p53 [32], in which transcription factor binding to DNA causes a conformation change which then leads to HAT binding. In our case, p300 acetylation may be an important post-DNA-binding event to stabilize the p300-Foxp3-DNA complex into a very stable state. DNA-bound K17R showed markedly reduced acetyl-bound Foxp3, suggesting that this lysine mutation causes altered conformation, which could affect its affinity for co-factors, including p300. Foxp3 is able to directly bind the promoters of various genes and up- or down-regulate their expression [38], [39]. In the current study, the altered expression of multiple Foxp3-dependent genes in CD4+ T cells transduced with Foxp3 K17R or K18R (Fig. 6) could be explained, at least in part, by different DNA binding caused by K17R or K18R.

The known association of permissive and repressive chromatin structures at targets that are differentially regulated [33] was also demonstrated in our studies (Fig. 4). Foxp3 may alter target gene promoter chromatin remodeling by recruiting HAT/coactivator or HDAC/corepressor complexes. The identification of a Foxp3 complex containing chromatin-remodeling factors, and data showing a Foxp3-Tip60-HDAC7 complex inhibits *IL-2* expression, support this idea [17]. Recent studies showed that the FKH domain mediates Foxp3 association with HDAC1, and that Foxp3 enhances HIV or inhibits IL-2 gene expression in human T cells by inhibiting HDAC1 activity [40]. Studies from our group and from others have shown that various HDAC enzymes contribute to regulation of Foxp3+ Treg production and function [16], [17], [41], [42], but little is known as to how Foxp3 regulates HDAC production and function. The current study shows that Foxp3 can regulate HDAC and HAT levels, and that specific lysines within the FKH play key and distinct roles in this process, pointing to a novel, but as yet unidentified, mechanism by which Foxp3 functions in Treg.

While Foxp3 is known to be acetylated by p300 and Tip60 [16], [17], no studies have yet mapped the precise lysine residue(s) within Foxp3 that must be acetylated for optimal Treg function, nor tested whether additional HAT enzymes also mediate Foxp3 acetylation. Our findings show that neither K17R nor K18R Foxp3 mutant had impaired p300-dependent acetylation, although both mutants led to impaired Treg suppression function. However, conclusions concerning whether K17 and/or K18 mediate Foxp3 acetylation cannot yet be made with confidence for at least 3 reasons. First, the lack of an anti-acetyl-Foxp3 Abs limits our ability to accurately assess Foxp3 acetylation at individual lysines. Second, intrinsic functional redundancy as a result of acetylation on different lysine residues may conceal the impairment caused by individual lysines. Third, we cannot exclude the possibility that K17R or K18R is a target of additional HAT enzymes.

In addition to acetylation, lysines are the targets of ubiquitination, sumoylation and methylation [43]. It is possible that disruption of one post-translational modification may affect additional post-translational modifications that thereby affect Foxp3 function. For example, acetylation of Foxp3 can prevent its ubiquitination and promote Foxp3 protein stability [18]. Since in our studies, replacement of K residues (K17, 18) with R, Q or E residues each impaired Treg suppressive function, a single molecular mechanism of suppression is unlikely and functional impairment may the result of multiple effects. As demonstrated in the case of Foxo1, acetylation and phosphorylation can cooperatively regulate the function of a transcription factor. Acetylation of Foxo1 at Lys-242, Lys-245, and Lys-262 alters its affinity for binding to target DNA, and increases its sensitivity for phosphorylation [43], [44]. Hence, studies are warranted to investigate how the various post-translational modifications, including phosphorylation, methylation or sumoylation, may form a dynamic and complex regulatory program to modulate Foxp3. Insights from such studies may ultimately provide new targets for therapeutic application of small molecules (HATi, HDACi or DNMTi) to precisely control and amplify or dampen Treg functions in vivo.

## Materials and Methods

### Ethics statement

Studies were approved by the Institutional Animal Care and Use Committee of The Children's Hospital of Philadelphia (approval numbers #2008-7-746 and #2010-6-561).

### Mice

We purchased Thy1.2+ C57BL/6 (B6) and B6/Thy1.1+ mice (Jackson Laboratory), and B6/Rag1−/− mice (Taconic). Mice were housed under pathogen-free conditions and used for experiments at 6–12 weeks of age.

### Antibodies and flow cytometry

Conjugated mAbs were purchased from eBioscience (eBioscience), flow cytometry was performed using a Cyan ADP Color flow cytometer (Beckman Coulter), and data were analyzed by FlowJo 8 software (TreeStar).

### Plasmids and mutagenesis

The indicated mouse Foxp3 cDNA lysines (K16, K17, K18, and K19) were substituted by arginine (R) or glutamine (Q) with the Quik-Change mutagenesis kit (Stratagene). After sequences were confirmed, fragments were recloned into MinR1 vector for retroviral expression. All plasmids used were sequenced (Napcore, The Children's Hospital of Philadelphia) to verify the correct configurations; sequences were analyzed using Lasergene software (DNASTAR).

### Retroviral transduction of primary T cells

Retroviruses were generated by cotransfection of WT MinR1-Foxp3 vector (WT-Foxp3), MinR1-Foxp3 vectors containing the indicated mutants, or parental MinR1 vector (EV), plus pCLeco (Invitrogen, CA) helper plasmid into the 293T-based Phoenix ecotropic packaging cell line using Lipfectamine 2000 (11668-019, Invitrogen). Virus containing supernatant was used to infect purified CD4+ T cells that were isolated with CD4+ T cell isolation kits (130-049-201, Miltenyi Biotec). Isolated CD4+ T cells were stimulated for 20 h using phorbol 12-myristate 13-acetate (PMA, 3 ng/mL), ionomycin (1 µM) and mouse IL-2 (10 U/mL, Roche). Activated T cells were infected with 48 h and 72 h viral supernatants harvested from transfected Phoenix cells, plus 10 U/mL mouse IL-2 and 4 µg/mL Polybrene (Sigma), followed by centrifugation for 90 min at 3,200 ramp. Cells cultured at 37° C with 5% CO_2_ for 2–3 d were used in the suppression assays or for RNA isolation.

### Treg suppression assays

Purified CD4+CD25− T cells and CD4+CD25+ Treg cells from wild-type C57BL/6 mice were isolated by CD4+CD25+ regulatory T cell isolation kit (130-091-041, Miltenyi Biotec). CD4+CD25− T cells were labeled with CFSE as effector cells and activated CD4+ T cells transduced with different Foxp3 constructs were utilized as Treg cells. Accordingly, 5×10^5^ CFSE-labeled effector T cells were stimulated with CD3 (5 µg/mL) in the presence of an equal number of irradiated syngeneic APC, isolated using kits from Miltenyi Biotec (130-049-101), and varying numbers of Treg cells [16]. Suppression of effector T cell proliferation was determined by flow cytometric analysis of CFSE dilution after 72 h.

### Homeostatic proliferation

Congenic Thy1.1+ CD4+CD25− T cells (1×10^6^), purified using a Treg isolation kit (130-091-041, Miltenyi Biotec), were mixed with 1×10^6^ Thy1.2+ CD4+ cells transduced with different Foxp3 constructs, and adoptively transferred to Rag1−/− mice [16]. Spleen and lymph nodes were isolated after 7 days and the total number of Thy1.1+ CD4+ T cells determined by flow cytometry.

### Quantitative PCR (qPCR)

Total RNA was extracted using an RNeasy Kit (Qiagen), cDNA was synthesized with TaqMan reverse transcription reagents (N808-0234, Applied Biosystems), and real-time PCR was performed using TaqMan Universal PCR Master Mix (4304437) and specific primers from Applied Biosystems.

### Transfection

293T cells were maintained (37 °C, 5% CO_2_) in RPMI-1640 plus 10% heat-inactivated FBS, penicillin and streptomycin. Cells were grown to 80–90% confluence, transfected with Foxp3 (12 µg), HA-p300 (12 µg) or empty vector using Lipofectamine 2000 (Invitrogen), harvested 48 h later, and cell lysates were prepared with using RIPA lysis and extraction buffer (Pierce).

### Nucleotide pull-down assay

Cell lysates (100 µg) from 293T cells transfected with empty vector, WT-Foxp3 or Foxp3 mutants in the absence or presence of p300 expression vectors were diluted in buffer (20 mM HEPES, 1.5 mM MgCl_2_, 0.2 mM EDTA, 1 mM DTT, 0.1% NP-40), and incubated overnight at 4 °C with 10 µg poly (deoxyinosinic-deoxycytidylic acid) (Roche) and 50 µl of streptavidin-agarose beads (Sigma) coated with biotinylated oligonucleotide containing putative Foxp3 binding sites: 5′-CAAGGTAAACAAGAGTAAACAAAGTC-3′ [30]. Beads were washed 5 times in dilution buffer, resuspended in 60 µl SDS sample loading buffer (BioRad), heated at 95 °C for 10 min, separated by SDS-PAGE, transferred to PVDF membranes and proteins detected using rat anti-mouse Foxp3 mAb or anti-acetyl-lysine Ab.

### Microarrays

RNA was prepared from CD4+ T cells transduced with EV, WT-Fopx3, Foxp3-K17R or Foxp3-K18R using an RNeasy Kit (Qiagen) and hybridized to Affymetrix GeneChip Mouse Genome 430A 2.0 arrays at the Nucleic Acid and Protein Core of The Children's Hospital of Philadelphia. Microarray data were analyzed by R/Bioconductor software (CBMi, The Children's Hospital of Philadelphia) and MAYDAY 2.12 software [45], assembled and processed in compliance with MIAME standards. Data were deposited in the NCBI Gene Expression Omnibus (GEO) database and are accessible through GEO Series accession number GSE27082 (http://www.ncbi.nlm.nih.gov/geo/query/acc.cgi?acc=GSE27082).

### Statistical analysis

Data were analyzed by GraphPad Prism software and are presented as mean ± SD; using a standard t-test; p<0.05 was considered significant and p<0.01 was considered highly significant.

## Supporting Information

Figure S1 K17R and K18R did not inhibit the activity of NFAT1 on NFAT∶AP-1 site. 293T cells were transfected with NFAP1-luciferase vector, NFAT1 and WT Foxp3 or K17R or K18R. Forty hours later, cells were stimulated with 1 mM ionomycin, 10 nM PMA, and 2 mM CaCl2 for 6 hours, dual luciferase activity was measured and results normalized to renilla.
(PPT)
