# Supplementary material for: Two Lysines in the Forkhead Domain of Foxp3 Are Key to T Regulatory Cell Function
Source: PLoS One. 2012 Jan 11;7(1):e29035. doi: 10.1371/journal.pone.0029035 (PMC3256141; doi:10.1371/journal.pone.0029035)
Supplement: Figure S1 — K17R and K18R did not inhibit the activity of NFAT1 on NFAT∶AP-1 site. 293T cells were transfected with NFAP1-luciferase vector, NFAT1 and WT Foxp3 or K17R or K18R. Forty hours later, cells were stimulated with 1 mM ionomycin, 10 nM PMA, and 2 mM CaCl2 for 6 hours, dual luciferase activity was measured and results normalized to renilla. (PPT) [file pone.0029035.s001.ppt]

## Slide 1
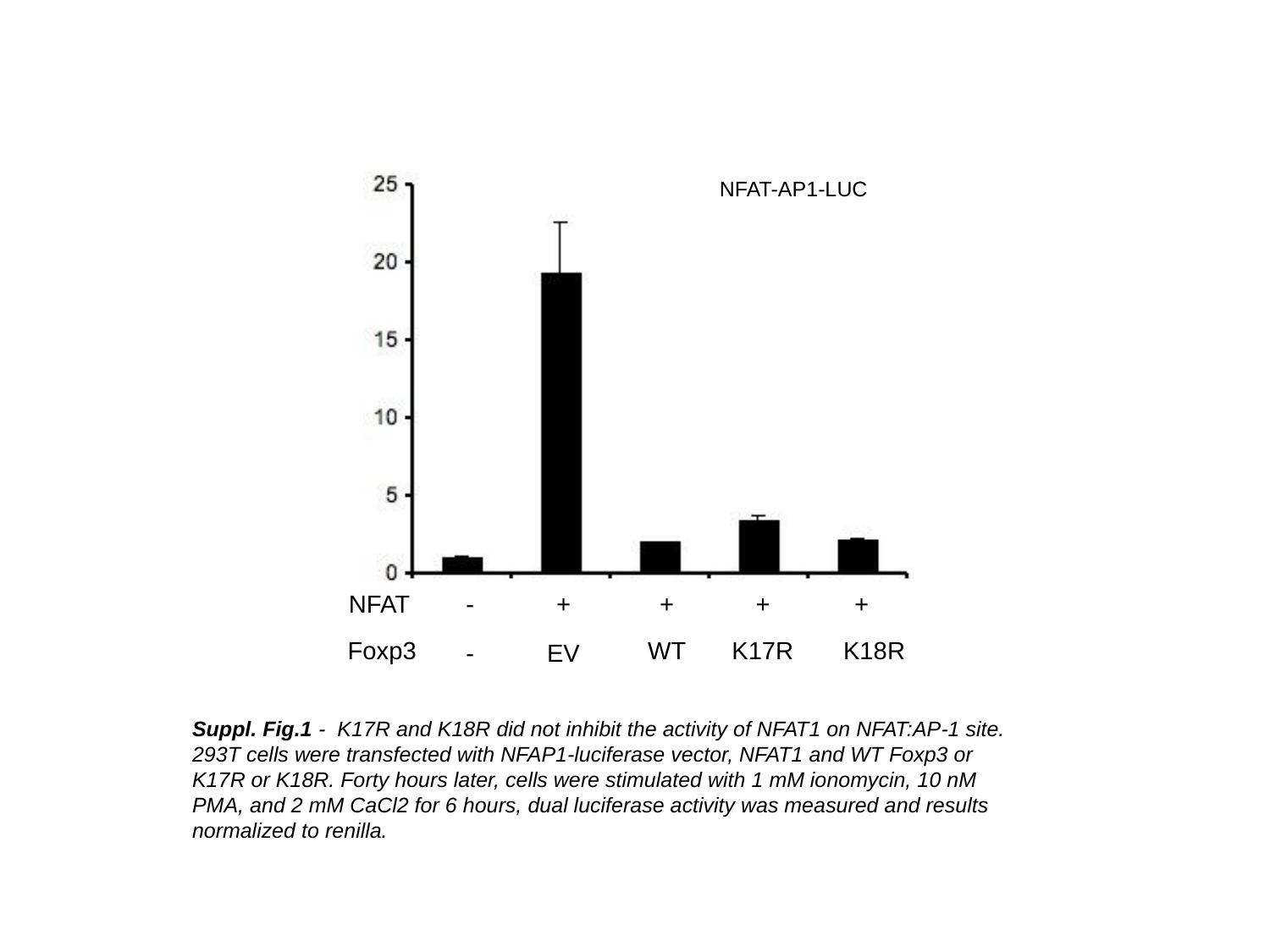

NFAT-AP1-LUC
NFAT
-
+
+
+
+
Foxp3
WT
K17R
K18R
-
EV
Suppl. Fig.1 - K17R and K18R did not inhibit the activity of NFAT1 on NFAT:AP-1 site. 293T cells were transfected with NFAP1-luciferase vector, NFAT1 and WT Foxp3 or K17R or K18R. Forty hours later, cells were stimulated with 1 mM ionomycin, 10 nM PMA, and 2 mM CaCl2 for 6 hours, dual luciferase activity was measured and results normalized to renilla.
